# Survey of work‐from‐home experiences among medical physicists in Southern California during and after the COVID‐19 pandemic

**DOI:** 10.1002/acm2.70523

**Published:** 2026-02-24

**Authors:** Xiaoyu Liu, Jennifer Zhang, David Hoffman, Varun Sehgal, Zhilei L. Shen, Chengyu Shi, X. Sharon Qi, Jing Cui, Amy S. Yu, Margaret Barker, Dan Ruan, Steve Goetsch

**Affiliations:** ^1^ Department of Radiation Oncology University of California, 3100 San Pablo Avenue San Francisco California USA; ^2^ Drexel University College of Medicine Philadelphia Pennsylvania USA; ^3^ Department of Radiation Medicine California Cancer Associates for Research and Excellence Encinitas California USA; ^4^ Department of Radiation Oncology University of California Irvine California USA; ^5^ Department of Radiation Oncology University of Southern California Los Angeles California USA; ^6^ Radiation Oncology City of Hope Irvine California USA; ^7^ Department of Radiation Oncology University of California, Los Angeles Los Angeles California USA; ^8^ Department of Radiation Oncology Cedars‐Sinai Medical Center Los Angeles California USA; ^9^ Department of Radiation Oncology Stanford University San Jose California USA; ^10^ Radiation Oncology MemorialCare Long Beach California USA; ^11^ Department of Radiation Oncology & Department of Bioengineering University of California, Los Angels Los Angeles California USA; ^12^ San Diego Gamma Knife Center, La Jolla San Diego California USA

**Keywords:** medical physicist, radiation oncology, the COVID‐19 pandemic, work‐from‐home

## Abstract

**Purpose:**

To evaluate the work‐from‐home (WFH) status of medical physicists in the American Association of Physicists in Medicine (AAPM) Southern California chapter (SCC) during and after the COVID‐19 pandemic.

**Materials and methods:**

An anonymous online survey was conducted through the SurveyMonkey platform and distributed to members of the AAPM SCC in January 2023. The 19 survey questions included eight multiple‐choice questions to collect demographic and background information, ten Likert‐scale items evaluating reasons for WFH, efficiency, flexibility, clinical coverage, collegial relationships, leadership trust, work hours, operating costs, employee satisfaction, and impacts on education, training, and research, plus one open‐ended question to obtain qualitative feedback. and the data was analyzed using both quantitative and qualitive methods. Quantitative data were summarized using descriptive statistics, while qualitative responses were thematically categorized.

**Results:**

At the end of a 5‐week collection period (January 3, 2023, to February 10, 2023), a total of 62 responses were received (33% response rate). Most respondents identified the COVID‐19 pandemic as the primary driver of WFH and reported that remote work increased job satisfaction, flexibility, and productivity while enabling timely completion of clinical duties. Reported concerns included reduced collegial relationships, limited trust in leadership, extended work beyond official hours, and decreased visibility relative to other clinical staff. Qualitative responses emphasized benefits such as reduced commuting and improved work–life balance, along with challenges for on‐site clinical duties and training. Hybrid work models were frequently identified as the most practical long‐term solution.

**Conclusions:**

This pilot study compared the WFH status among medical physicists from AAPM SCC during and after the COVID‐19 pandemic and highlights both advantages and limitations of WFH for medical physicists. WFH offers notable benefits for medical physicists, including enhanced flexibility and satisfaction. However, essential clinical responsibilities require on‐site presence. These findings support the development of tailored hybrid work models and inform future workforce strategies. Expansion to a national survey is planned to further evaluate WFH practices within the medical physics community.

## 1 INTRODUCTION

The COVID‐19 pandemic significantly disrupted healthcare systems worldwide, necessitating rapid adoption of new workflows and enhanced safety protocols to continue providing quality care to patients while minimizing infection risk to both patients and staff. The field of radiation oncology was no exception.^1^ The COVID‐19′s global reach profoundly affected radiation therapy practices worldwide, including in the United States.^2^ The pandemic reshaped clinical workflows,^3^, ^4^, ^5^ educational models,^6^ and professional engagement, with long‐term implications for the financial health of scientific societies. For instance, the European society for radiotherapy and oncology outlined key focus areas for adapting to pandemic‐driven challenges.^7^ These adjustments—ranging from the implementation of remote work and patient education to the prioritization of infection control measures and resource optimization—were essential to sustaining cancer care while protecting healthcare workers and patients.

Earlier studies^2^, ^8^ have primarily relied on experiences of individual radiation therapy center's practice changes in response to the COVID‐19 pandemic. Other articles^9^, ^10^ discuss the impacts that the COVID‐19 pandemic had on patient care and clinical practices of medical physics, identifying work‐from‐home (WFH) as one of the most commonly adopted changes for medical physicists.^11^, ^12^ One research group undertook a pilot study^13^ to assess the practice changes during the COVID‐19 pandemic among a cohort of 61 radiation oncology centers in California. The survey indicates that the most significant practice changes reported by respondents can be categorized into three main areas: the implementation of new department policies; adoption of new hybrid work models, and changes in patient treatment plans, including increased use of hypofractionation, which has substantially reduced patients’ treatment duration and has continued to be increasingly adopted in clinical practice after the COVID‐19 pandemic.

Another study^14^ analyzed the correlations among WFH practices, job stress, burnout, and job satisfaction. While some tasks, such as treatment planning and chart checks proved compatible with remote execution, others, including machine quality assurance, brachytherapy procedures, remained on‐site. Some of these changes may be temporary, other changes have added unanticipated benefits to many organizations and will likely remain.

Despite growing interest in hybrid work models, few studies have specifically examined the long‐term viability and perceptions of WFH among medical physicists. Existing literature has addressed general practice changes and well‐being, but a focused evaluation of WFH practices within this professional group is lacking.

This study aims to fill that gap by surveying members of the AAPM SCC to assess their WFH experiences during and after the COVID‐19 pandemic. The objectives are to:

1. Quantify the extent and nature of WFH adoption among medical physicists.
2. Evaluate perceived impacts on workflow efficiency, clinical coverage, professional relationships, and job satisfaction.
3. Identify challenges and benefits associated with remote work.
4. Explore preferences for future work models, including hybrid arrangements.

These findings have the potential to improve the quality of patient's care, medical physicists’ work–life balance, along with reducing operational costs at radiation oncology centers throughout the country.

## 2 MATERIALS AND METHODS

A 19‐question survey was developed using SurveyMonkey and distributed via email to members of the AAPM SCC. The survey was open from January 3 to February 10, 2023, and participation was voluntary and anonymous.

The survey was structured into three sections (Appendix)

- Section 1 (Questions 1–8): These multiple‐choice questions were designed to collect demographic and background information, including age, gender, employer, job title, number of patients treated per day, WFH schedule and patient load per day changes during and after the COVID‐19 pandemic. Responses to Questions 1–8 are summarized in Table 1.
- Section 2 (Questions 9–18): These items were rated on a 0–10 Likert scale. Respondents were asked to assess various aspects related to WFH. For example, if participants believed that COVID‐19 was the main reason for WFH, they would score 10; if they did not believe it was a reason, they would score 0; and if they believed it may have been the reason, they would score 5. Using similar scoring, participants evaluated the impact of WFH on workflow efficiency and flexibility, the ability of medical physicists to complete clinical duties remotely, perceived effects on interpersonal relationships, trust and respect among colleagues, trust in leadership, support for education, training, and research, changes in working hours beyond official schedules, potential cost savings for employers, and the overall impact of WFH on employee satisfaction. Responses to Questions 9–18 are summarized in Table 2.
- Section 3 (Question 19): This was an open‐ended question that invited respondents to share additional comments or experiences related to WFH.

**TABLE 1:** Demographic and work‐from‐home characteristics of survey respondents.

Characteristic	Category	Percentage (%)
Q1 Age (years)	< 30	5
	31–40	23
	41–50	40
	51–60	18
	> 60	14
Q2 Gender identity	Female	34
	Male	66
	Non‐binary	0
	Prefer not to say	0
Q3 Employer type	University medical center	46
	Community hospital	32
	Private clinic	20
	Self‐employed	2
Q4 Job title	Junior medical physicist	2
	Mid‐career medical physicist	58
	Senior medical physicist	27
	Chief medical physicist	14
Q5 Patients treated per day at center	< 50	32
	51–100	20
	101–150	19
	151–200	17
	> 200	12
Q6 WFH availability during COVID‐19 pandemic (03/2020–06/2021)	Entirely WFH	8
	Partially WFH	66
	Not able to WFH	26
Q7 Change in patient load during pandemic	No change	58
	Decreased	12
	Increased	30
Q 8 WFH status after California reopening (post 06/2021)	Entirely WFH	2
	Partially WFH	49
	Not able to WFH	38
	Never able to WFH	11

Abbreviation: WFH, work from home.

**TABLE 2:** Summary of responses to Likert‐scale questions (Questions 9–18).

Survey item	Mean	Standard deviation
Q9 COVID‐19 pandemic was the primary reason for WFH	8.5	2.4
Q10 WFH or partial WFH improved workflow efficiency	6.8	2.8
Q11WFH or partial WFH increased work flexibility	7.4	2.7
Q12WFH or partial WFH allowed timely completion of clinical duties	7.2	2.8
Q13 WFH or partial WFH improved collegial relationships, trust, and respect	5.5	2.9
Q14 WFH or partial WFH enhanced trust in leadership	5.9	2.8
Q15 WFH improved support for education, training, and/or research	6.1	3.2
Q16 WFH increased working hours outside official work hours	5.8	2.8
Q17 WFH allowed employer cost savings without sacrificing productivity	6.7	3.0
Q18 Overall impact of WFH on employee satisfaction	8.2	2.2

*Note*: Responses were rated on a 0–10 Likert scale, with higher scores indicating stronger agreement.

Abbreviation: WFH, work from home.

Data were analyzed using both qualitative and quantitative methods, quantitative data were analyzed using descriptive statistics, including means and standard deviations. Qualitative responses were reviewed and thematically categorized to identify recurring patterns and insights. Statistical analysis was performed using SAS Enterprise Guide (version 9.1).

## 3 RESULTS

### 3.1 Survey response rate and demographics

Out of 188 distributed surveys to AAPM full members who work in Southern California within the AAPM SCC, 62 responses were received, yielding a response rate of 33% This rate exceeds that of previous related studies, including the 2012 ASTRO comprehensive workforce study^15^ (18%), and a previous survey on medical physicist well‐being in California (29%).^14^

As shown in Table 1, respondents’ age was distributed as follows: 5% under 30 years, 23% aged 31–40 years, 40% aged 41–50 years, 18% aged 51–60 years, and 14% over 60 years. The gender distribution was 34% female and 66% male. Employment included 46% at university medical centers, 32% at community hospitals and 20% at private clinics. Job titles were 2% junior medical physicists, 58% mid‐career medical physicists, 27% senior medical physicists, and 14% chief medical physicists. Regarding patient load, 32% reported fewer than 50 patients treated per day, 20% treated 51–100 patients, 19% treated 101–150 patients, 17% treated 151–200 patients and 12% treated over 250 patients per day. During the pandemic, 58% reported no change in patient load, 12% reported a decrease, and 30.0% reported an increase post the pandemic.

### 3.2 WFH status

Based on Question 6 and 8 (Table 1), during the COVID‐19 pandemic (March 2020–June 2021), 8% of respondents worked entirely from home, 66% worked partially from home, and 26% were unable to WFH. After California's reopening post June 2021, only 2% of respondents continued to work entirely from home. 49% of the respondents worked partially from home, and 38% were unable to work remotely, including 11% who never worked from home. These results indicate a shift back towards on‐site work following the COVID‐19 pandemic.

### 3.3 Quantitative analysis

Responses to Likert‐scale questions (0–10 scale) are reported in Table 2, in which WFH was assessed across multiple domains, including reasons for WFH, workflow efficiency and flexibility, ability to complete clinical duties remotely, interpersonal relationships, trust and respect among colleagues, trust in leadership, support for education, training, and research, changes in working hours beyond official schedules, potential cost savings for employers, and overall employee satisfaction.

Most respondents agreed that COVID‐19 was the main reason for adopting WFH (mean = 8.5) and that it positively affected job satisfaction (mean = 8.2) and flexibility (mean = 7.4), while allowing timely completion of clinical duties (mean = 7.2). However, views were more variable regarding its impact on education, training, and research (SD = 3.2), and WFH was generally not seen as maintaining strong collegial relationships or trust (SD = 2.9).

In summary, the table revealed that respondents strongly agreed that the COVID‐19 pandemic was the main driver for WFH adoption and that WFH positively impacted job satisfaction and flexibility. However, perceptions were more mixed regarding interpersonal relationships, leadership trust, and educational support.

### 3.4 Qualitative analysis

Open‐ended responses revealed nuanced perspectives:

Perceived benefits included increased efficiency and productivity due to fewer workplace interruptions. Successful remote execution of tasks such as treatment planning, chart checks, stereotactic radiosurgery planning, and quality assurance (QA) review. Improved work–life balance reduced commuting time, and greater flexibility for childcare.

Responses also identified major challenges in:

- Essential clinical duties (e.g., patient‐specific QA, machine QA, brachytherapy) require on‐site presence.
- Concerns about fairness and visibility, especially compared to other clinical staff (radiation therapists, nurses, etc.) who remained on‐site.
- Reduced collaboration and mentorship opportunities, particularly for new staff and trainees.
- Home environments are not always conducive to focused work due to family‐related distractions.

Overall, many respondents endorsed hybrid work arrangements, indicating that maintaining at least one physicist on‐site while others work remotely provides a practical operational balance. Some respondents also advocated flexible scheduling models, such as European‐style four‐day workweeks.

## 4 DISCUSSION

The ongoing debate^12^ over WFH models continues, as some healthcare organizations move toward mandatory on‐site work in the post‐pandemic era. Our study sheds light on how medical physicists in Southern California experience this shift, finding a consensus that WFH improves flexibility, work–life balance, and overall job satisfaction. However, a limitation is also observed: remote work may not seem to foster strong professional relationships, trust, and collegial respect that are built through in‐person interaction.

This range of opinions highlights a crucial point: there is no universal model for remote work by medical physicists. While certain tasks are well‐suited for a home office, essential clinical responsibilities—such as machine QA and brachytherapy procedures—will always require a physical presence. The most pragmatic solution appears to be a hybrid model that balances these needs. However, the successful implementation of such a model is not a one‐size‐fits‐all solution. It must be tailored to specific institutional factors.

Larger departments with a greater number of physicists may find it easier to implement a rotating hybrid schedule, ensuring that at least one physicist is always on‐site. Smaller departments may lack this flexibility. Additionally, practices with high patient loads or a focus on complex treatments like stereotactic body radiation therapy and brachytherapy will require more consistent on‐site coverage. The existing culture of trust and collaboration within a department will also heavily influence how well a remote or hybrid model is adopted and whether it succeeds.

In conclusion, WFH arrangements for medical physicists appear most effective when implemented in a hybrid manner that accommodates essential on‐site responsibilities while preserving flexibility for tasks that can be performed remotely

It's important to acknowledge the scope of this study, which is limited to the perspective of medical physicists. Any decision to adopt WFH or hybrid schedules must also consider the roles and needs of the entire clinical team, including radiation oncologists, therapists, nurses, dosimetrists, and administrators. This holistic approach is essential for maintaining seamless patient care and a cohesive practice environment.

The findings are constrained by both geographic and demographic factors. To build upon these results, a more comprehensive, nationwide survey would help validate and extend the findings by providing a deeper understanding of medical physicists' experiences with WFH arrangements and by assessing their broader impacts on productivity, professional practice, and balance of work–life across the entire field.

## 5 CONCLUSION

This pilot study offers valuables insights into the WFH status of medical physicists in Southern California and its multifaceted impact on their professional role. The COVID‐19 pandemic significantly accelerated the adoption of WFH models. While WFH offers benefits such as increased efficiency and productivity, its effects on interpersonal relationships, leadership trust, and especially educational support are more nuanced and mixed. While workforce composition and culture, technology and nature of pandemic are dynamic, the findings of this study highlight several critical considerations for preparedness and response. Key factors include fostering collegial interaction, mitigating peer tension, maintaining a sense of community, and supporting training and education during WFH arrangements. In hybrid setting, strategies for staggered WFH schedules should be optimized to promote fairness. And above all, it is essential to remain mindful of workforce well‐being and to provide open communication and support channels. Future research will involve a national survey to validate and extent these findings, with the goal of identifying optimal work models that enhance medical physicists’ work–life balance while ensuring patient safety and maintaining high quality clinical care.

## AUTHOR CONTRIBUTIONS

Conception and design of the study: Xiaoyu Liu, Jennifer Zhang, Steve Goetsch. Data collection and analysis: David Hoffman, Jennifer Zhang, Xiaoyu Liu. Writing and revising: Xiaoyu Liu, Jennifer Zhang, Dan Ruan, David Hoffman. Varun Sehgal, Zhilei L. Shen, Chengyu Shi, X. Sharon Qi, Jin Cui, Amy S. Yu, Margart Barker, Steve Goetsch. The final manuscript was approved by all the authors. All the authors agreed to be accountable for all aspects of the work in ensuring that the questions related to the accuracy or integrity of any part of the work are appropriately investigated and resolved.

## CONFLICT OF INTEREST STATEMENT

All the authors declare no conflict of interest.

## Supporting information



Supporting Information
