# Supplementary material for: Survey of work‐from‐home experiences among medical physicists in Southern California during and after the COVID‐19 pandemic
Source: J Appl Clin Med Phys. 2026 Feb 24;27(3):e70523. doi: 10.1002/acm2.70523 (PMC12931420; doi:10.1002/acm2.70523)
Supplement: Supplementary file 1 — Supporting Information [file ACM2-27-e70523-s001.docx]

**Appendix**

**Medical Physicist Work from Home Status Survey Questions**

1. **What is your age?**

Under 30 years old

31-40 years old

41-50 years old

51-60 years old

Over 60 years old

1. **To which gender identity do you most identify with?**

Female

Male

Non-binary

Prefer not to say

1. **Who is your employer?**

University Medical Center

Community Hospital

Private Clinic

Self-Employed

Other (please specify)

1. **What is your job title?**

Medical physicist

Chief medical physicist

Assistant Professor

Associate Professor

Professor

Other (please specify)

1. **About** **How many patients does your center treat per day?**

Under 50

51-100

101-150

151-200

More than 200

1. **Were you able to work from home anytime from the start of the COVID-19 pandemic until California official reopened (03/2020-06/2021)?**

Yes, I was entirely able to WFH

Yes, I was partially WFH

No, I was not able to WFH

1. **Has your center’s patient load remained the same as compared to before the COVID-19 pandemic (i.e. before March 2020)?**

Yes, patient load remain the same

No, patient load decreased

No, patient load increased

1. **Do you still work from home (WFH), or partially WFH after California’s reopening (i.e. after June 2021)?**

Yes, I am entirely able to WFH

Yes, I am partially able to WFH

No, I am not able to WFH

I was never able to work from home

1. **Do you think COVID-19 pandemic is the main reason why your department start WFH/Partially WFH?**

**0** 1 2 3 4 **5** 6 7 8 9 **10**

0=Not at all 5=Somewhat 10=Extremely

**10. In your opinion, do you think WFH/Partially WFH is a more efficient workflow for medical physicists’ productivity than completely working on-site?**

0 1 2 3 4 5 6 7 8 9 10

0=Not at all 5= Somewhat 10=Extremely

**11. In your opinion, do you think WFH/Partially WFH provides more flexibility to accomplish work tasks, such as treatment planning, machine QA, than completely working on-site?**

0 1 2 3 4 5 6 7 8 9 10

0=Not at all 5=Somewhat 10=Extremely

**12. Do you think WFH/Partially WFH can still cover clinical works on time for medical physicists?**

0 1 2 3 4 5 6 7 8 9 10

0=Not at all 5=Somewhat 10=Extremely

**13. Do you think WFH/Partially WFH is more connected with close relationships, trust and respect among colleagues?**

0 1 2 3 4 5 6 7 8 9 10

0=Not at all 5=Somewhat 10=Extremely

**14. Do you think WFH/Partial WFH can enhance trust in the leadership?**

1. 1 2 3 4 5 6 7 8 9 10

0=Not at all 5=Somewhat 10=Extremely

**15. Do you think WFH/Partial WFH is more convenient for education, training and/or research for medical physicists?**

0 1 2 3 4 5 6 7 8 9 10

0=Not at all 5= Somewhat 10=Extremely

**16. Do you think WFH/Partial WFH can add more working hours for medical physicists outside of official work hours?**

0 1 2 3 4 5 6 7 8 9 10

0=Not at all 5=Somewhat 10=Extremely

**17. Do you think WFH/Partial WFH is good for employer to allow significant cost saving in infrastructure and business costs without sacrificing employee productivity?**

0 1 2 3 4 5 6 7 8 9 10

0=Not at all 5=Somewhat 10=Extremely

**18. What is the impact of WFH on employee satisfaction, including the elimination of commute times, decreasing work expenses, and supporting a better work-life balance?**

0 1 2 3 4 5 6 7 8 9 10

0=Not at all 5=Somewhat 10=Extremely

**19. Are there any additional comments/experiences you would like to share? Please comment below:**
